# Mixed Transition-Metal Oxides on Reduced Graphene Oxide as a Selective Catalyst for Alkaline Oxygen Reduction

**DOI:** 10.1021/acsomega.3c00615

**Published:** 2023-03-15

**Authors:** Sigrid Wolf, Michaela Roschger, Boštjan Genorio, Daniel Garstenauer, Viktor Hacker

**Affiliations:** † Institute of Chemical Engineering and Environmental Technology, Graz University of Technology, Inffeldgasse 25/C, 8010 Graz, Austria; ‡ Faculty of Chemistry and Chemical Technology, University of Ljubljana, Večna pot 113, 1000 Ljubljana, Slovenia

## Abstract

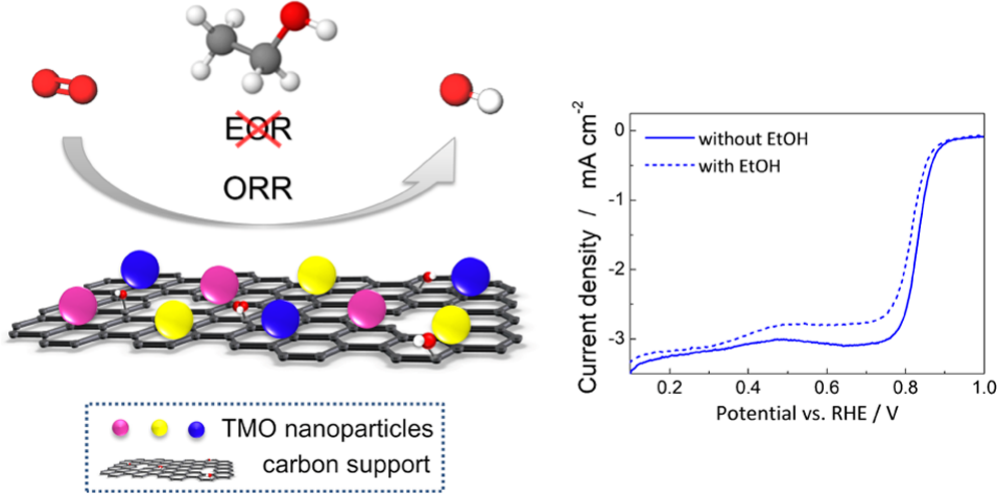

The development of highly efficient, stable, and selective non-precious-metal catalysts for the oxygen reduction reaction (ORR) in alkaline fuel cell applications is essential. A novel nanocomposite of zinc- and cerium-modified cobalt-manganese oxide on reduced graphene oxide mixed with Vulcan carbon (ZnCe-CMO/rGO-VC) was prepared. Physicochemical characterization reveals uniform distribution of nanoparticles strongly anchored on the carbon support resulting in a high specific surface area with abundant active sites. Electrochemical analyses demonstrate a high selectivity in the presence of ethanol compared to commercial Pt/C and excellent ORR activity and stability with a limiting current density of −3.07 mA cm^–2^, onset and half-wave potentials of 0.91 and 0.83 V vs reversible hydrogen reference electrode (RHE), respectively, a high electron transfer number, and an outstanding stability of 91%. Such a catalyst could be an efficient and cost-effective alternative to modern noble-metal ORR catalysts in alkaline media.

## Introduction

Increasing energy consumption and the associated environmental impact of fossil fuel use make the shift to renewable energy technologies such as fuel cells inevitable. Alkaline direct ethanol fuel cells (ADEFCs) are of particular interest because ethanol fuel is sustainable, easy to handle, and has a high energy density.^1,2^ Research for ADEFCs is mainly concerned with the development of new anode catalysts since the ethanol oxidation reaction (EOR) at the anode is strongly hindered.^3^ However, the oxygen reduction reaction (ORR) at the cathode is also essential as it suffers from sluggish kinetics. The ORR can proceed via the direct four-electron pathway (eq 1), which is strongly preferred, or via the two-electron pathway (eqs 2 and 3), which involves an intermediate step of HO_2_^–^ production lowering the overall ORR performance.^4^
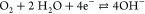
1
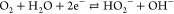
2
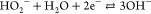
3

Precious-metal catalysts (e.g., Pt/C) are effective for the reduction of O_2_ via the four-electron pathway but are associated with high costs and susceptibility to poisoning.^5,6^ In ADEFCs, ethanol crossover from the anode to the cathode is a very important issue that also needs to be tackled. Since Pt/C also exhibits a high activity for the EOR, the ORR and EOR can occur simultaneously when ethanol is present at the cathode, resulting in mixed potentials and thereby decreasing the overall cell efficiency.^7^ Non-precious-metal catalysts, including various transition-metal oxides (TMOs), show great potential for overcoming these challenges. They exhibit high tolerance to ethanol crossover, are cost-efficient, and show excellent ORR activity and stability in alkaline media.^5,7^ Spinels such as CoMn_2_O_4_ (CMO), which are suitable due to their multiple valences, have been well studied as ORR catalysts.^8−10^ Other metal oxides such as CeO_2_, which draws particular attention due to its Ce^3+^/Ce^4+^ redox couple and abundant oxygen vacancies, or ZnO (presents high chemical stability and nontoxicity) are also being increasingly explored.^11,12^ However, the poor conductivity and small surface area of TMOs limit their application; hence, they are often deposited on conductive carbon materials, such as reduced graphene oxide (rGO). Sun et al.,^13^ for example, have prepared a CeO_2_/rGO composite being tolerant to alcohol and having high catalytic ORR activity and stability generated by the 4f orbit of cerium and the electronic interactions between CeO_2_ and rGO. In another study, Du et al.^14^ described that CoMn_2_O_4_/rGO is more active and stable than Pt/C in alkaline media, which is due to small particle size and good distribution induced by rGO. ZnO/rGO has also been explored by Yu et al.^12^ to be a promising non-precious-metal cathode in alkaline fuel cells.

In addition to the use of carbon substrates, the development of active ORR catalysts can also be promoted by mixing different metal oxides to benefit from the individual properties and their synergistic effects. Zhong et al.^15^ reported that the addition of CeO_2_ to CoO*_x_* on rGO can increase its ORR performance since CeO_2_ acts as an “oxygen buffer” and thus facilitates O_2_ release/storage. In a paper by Liu et al.,^16^ a ZnO/ZnCo_2_O_4_/C@rGO composite was described as highly active and stable in alkaline conditions and as tolerant to alcohol crossover.

To further enhance the performance of ORR based on TMO electrocatalysts, we synthesized and tested zinc- and cerium-modified cobalt-manganese oxide catalysts on rGO and Vulcan carbon supports for the first time. The electrocatalytic activity and stability of the nanocomposite toward ORR in alkaline media were investigated, and the susceptibility to ethanol poisoning was studied. The synergistic effects between the individual components and the high selectivity provide excellent performance of the catalyst and imply the applicability for ORR in alkaline fuel cell applications.

## Materials and Methods

### Materials

The following chemicals were used for material preparation and electrochemical analysis: graphite (Timrex KS44; Imreys, Bodio, Switzerland), carbon black (Vulcan carbon XC72R; Cabot Corp., Boston, Massachusetts), hydrazine hydrate (reagent grade; Sigma-Aldrich, Darmstadt, Germany), cobalt(II) nitrate hexahydrate (99.999% trace metals basis; Sigma-Aldrich, Darmstadt, Germany), zinc(II) nitrate hexahydrate (>99%; Honeywell Fluka, Charlotte, North Carolina), cerium(III) nitrate hexahydrate (99.5%; Alfa Aesar, Haverhill, Massachusetts), manganese(II) nitrate tetrahydrate (98%; Alfa Aesar, Haverhill, Massachusetts), ammonium hydroxide solution (30–33%; Honeywell, Charlotte, North Carolina), 2-propanol (≥99.9%, UV/IR-grade; Carl Roth, Karlsruhe, Germany), potassium hydroxide (1.0 M Fixanal 1 L Ampoule; Merck, Darmstadt, Germany), ethanol (99.9% p.a.; Carl Roth, Karlsruhe, Germany), Nafion solution (5 wt % in water; Quintech, Göppingen, Germany), alumina suspension (0.05 μm; MasterPrep Bühler, Lake Bluff, Illinois), and Pt/C (30 wt % on Vulcan; De Nora North America, New Jersey). Ultrapure water (∼18 MΩ cm) was used in all experiments.

### Catalyst Preparation

The composite catalyst was prepared by the deposition of the transition-metal oxides on the carbon support material via a facile synthesis method.^17−19^ First, graphene oxide (GO), which was produced from graphite powder using the Hummers method, was chemically reduced with hydrazine hydrate at 105 °C for 24 h to prepare rGO. The resulting material was filtered, washed with hot water and ethanol, and finally dried under ambient conditions (24 h) and vacuum (80 °C, overnight). Then, 130 mg of the prepared rGO was mixed with 32 mg of Vulcan carbon XC72R (VC) in 5 mL of ultrapure water and 1 mL of 2-propanol. Thereafter, transition-metal nitrate hexahydrates of Zn (36 mg), Ce (43 mg), and Co (41 mg) were dissolved in 15 mL of ultrapure water and added to the rGO/VC mixture. After 30 min of ultrasonication, 4 mL of an aqueous ammonium hydroxide solution was dropped into the dispersion, followed by another 30 min of ultrasonication. Manganese nitrate tetrahydrate (76 mg) was then dissolved in 5 mL of ultrapure water and slowly added to the mixture. Finally, ultrasonication was carried out for another 60 min, and then, the dispersion was kept at 180 °C overnight, and the solvent was evaporated to gain the ZnCe-CMO/rGO-VC catalyst.

### Physicochemical Characterization

Comprehensive physicochemical characterization was carried out to analyze the structure, chemical composition, morphology, specific surface area (SSA), and thermogravimetric behavior of the composite catalyst. X-ray diffractometry (XRD) with Cu Kα_1_ (λ = 0.15406 nm) radiation in a 2θ range between 10 and 60° (0.02° min^–1^ 2θ step size) was performed on a PANalytical X’Pert PRO MPD (Almelo, Netherlands) X-ray diffractometer utilizing a fully opened X’Celerator detector. The metal content was determined by an Agilent Technologies 7900 (Palo Alto, California) inductively coupled plasma mass spectrometer (ICP-MS) using high-purity Ar gas (5.0) at a flow rate of 15 L min^–1^. Data were acquired and analyzed with MassHunter 4.4. software. Scanning electron microscopy (SEM) studies of the sample adhered on a conductive carbon tape were conducted on a Zeiss ULTRA plus (Jena, Germany) field emission scanning electron microscope (2 kV, WD = 6 mm, SE2, and inlens detector). The Brunauer–Emmett–Teller (BET) SSA was evaluated after outgassing the samples at 200 °C for 4 h by recording N_2_ adsorption/desorption isotherms (relative pressure range from 0.01 to 0.99) on a ASAP 2020 Micromertics (Norcross, Georgia) system. Thermogravimetric analysis (TGA) was carried out with a Netsch 449 F3 Jupiter (Selb, Germany) analyzer. The samples were heated in a temperature range from 30 to 600 °C in O_2_/Ar (50 mL min^–1^) with a heating rate of 10 K min^–1^. Simultaneously, a MS 403C Aëolos (Netzsch, Selb, Germany) with a SEM Chenneltron detector was used to analyze the evolved gases by mass spectroscopy (MS) at 220 °C and 2 × 10^–5^ bar.

### Electrochemical Characterization

The electrochemical properties of the ZnCe-CMO/rGO-VC composite were investigated by means of thin-film rotating disk electrode (RDE) experiments. Cyclic voltammetry (CV), linear sweep voltammetry (LSV), and chronoamperometry (CA) measurements were performed using a Reference 600 potentiostat/galvanostat/ZRA and a software from GAMRY Instruments (Warminster, Pennsylvania). In a standard three-electrode system, a platinized titanium rod (Bank Elektronik - Intelligent controls GmbH, Pohlheim, Germany) as a counter electrode, a reversible hydrogen reference electrode (RHE, HydroFlex, Gaskatel, Kassel, Germany), and the catalyst sample-modified glassy carbon (GC) working electrode with an area of 0.196 cm^2^ were installed. The working electrode was prepared by pipetting 10 μL of a catalyst ink (4.2 mg catalyst powder, 490 μL H_2_O, 490 μL 2-propanol, and 20 μL Nafion) onto an Al_2_O_3_-polished GC disk. The solvent was evaporated by rotation at 700 rpm under ambient conditions, and a final loading of 210 μg cm^–2^ was achieved. The CV and LSV tests were conducted at 30 °C in N_2_- or O_2_-saturated 1 M KOH electrolyte solution, respectively, and were similarly repeated in 1 M KOH/1 M EtOH to examine the influence of the presence of ethanol on the redox processes and the ORR activity. The voltammograms were recorded at a scan rate of 10 mV s^–1^ in a potential range from 0.1 to 1.0 V vs RHE. LSV was performed at different rotation rates (400, 600, 900, 1200, 1600, and 2000 rpm), and the curves are corrected by the CV for interpretation of the results. CA measurement was carried out in O_2_-saturated 1 M KOH electrolyte solution at 1000 rpm by applying a potential of 0.4 V vs RHE for 3600 s. Subsequently, 2.92 mL of EtOH were added, and the stability in presence of ethanol was analyzed for further 1000 s.

## Results and Discussion

### Physicochemical Characterization of the Composite Catalyst

**Figure 1:**
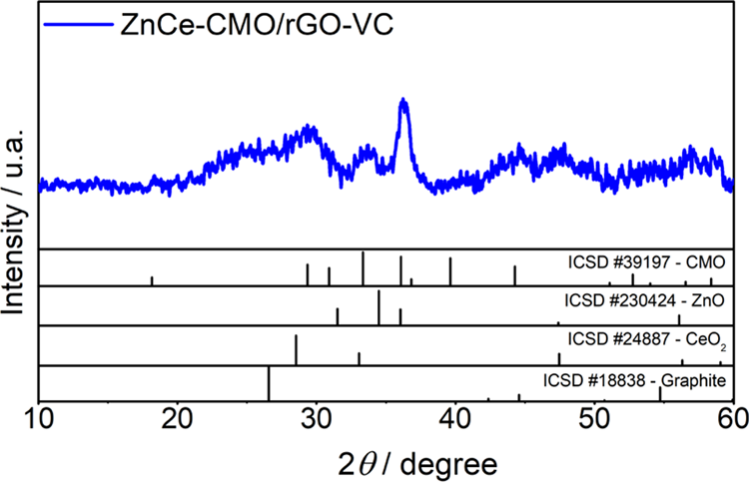
XRD pattern of ZnCe-CMO/rGO-VC compared with CMO, ZnO, CeO_2_, and graphite standard data from ICSD.

The XRD pattern of ZnCe-CMO/rGO-VC is presented in Figure 1, and the standard data of CMO (ICSD #39197), ZnO (ICSD #230424), CeO_2_ (ICSD #24887), and graphite (ICSD #18838) are added at the bottom for analyzing the crystalline nature of the composite material.

The results reveal the coexistence of the carbon support and very small metal oxide particles, indicated by the broad peaks. The diffraction peaks at 2θ of approx. 25 and 43° are related to the (002) and (101) planes of the graphite-like structure of the successfully prepared rGO by deoxygenation of GO.^18,20^ All other peaks can be indexed to the crystal structures of the CMO spinel, CeO_2_, and ZnO, as the comparison with the ICSD standard data reveals. The average crystallite size of the metal oxide nanoparticles was estimated to be approx. 8 nm using the Scherrer equation *D* = 0.9λ/β cos θ, where *D* is the mean size of the particles in nm, λ is the X-ray wavelength (0.15406 nm), β is the peak width at half-height in radians, and θ is Bragg’s angle.^13,21^

**Figure 2:**
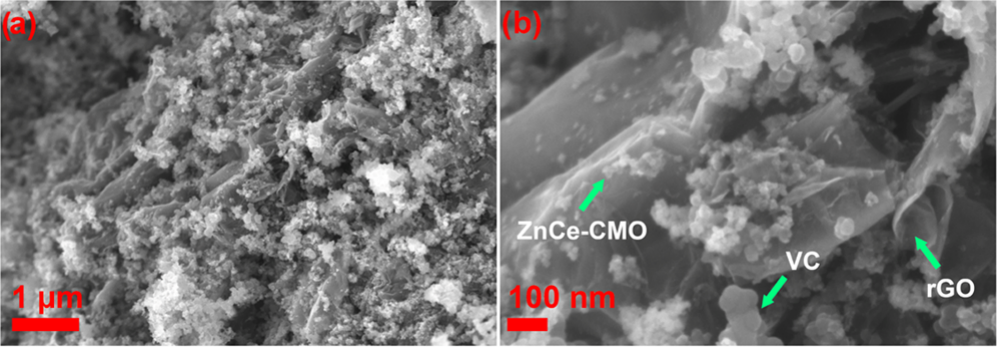
SEM images of ZnCe-CMO/rGO-VC at (a) lower and (b) higher magnification.

The surface morphology and particle size distribution of ZnCe-CMO/rGO-VC were examined by SEM analysis. As can be seen in Figure 2, the SEM images at different magnifications display a two-dimensional wrinkled sheet-like structure typical for the rGO material^12,16,22^ and a few spherical carbon particles (<100 nm) attributed to VC. Even though rGO is already considered a recognized carbon support material, the rGO sheets tend to agglomerate and restack due to π–π interactions, resulting in a decreased surface area and reduced electrical conductivity and thus an overall drop in catalyst activity. Therefore, a small amount of VC particles was added as a spacer to prevent restacking of the rGO sheets.^23^ In addition, metal oxide particles distributed on the carbon support material, which were estimated using the Scherrer equation to have a crystallite size in the range of approx. 8 nm, can be detected, as shown in the higher-magnification image (Figure 2b). A strong contact of the ZnCe-CMO active material with the rGO sheets suggesting a spherical particle shape with some agglomerates is indicated. The small particle size and anchoring of the metal oxide particles are ensured by the strong C–O–metal bridge created by the remaining oxygen-containing functionalities of the rGO. Abundant active sites are therefore generated, which can have a positive effect on the activity and especially the stability of the catalyst.^12,16^

**Figure 3:**
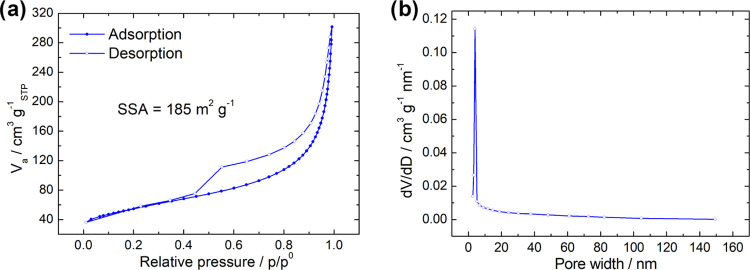
(a) Nitrogen adsorption/desorption isotherms and (b) BJH desorption pore size distribution of ZnCe-CMO/rGO-VC.

Nitrogen adsorption–desorption isotherms in a relative pressure range from 0.01 to 0.99 were recorded, and the SSA of ZnCe-CMO/rGO-VC was evaluated using the BET method (Figure 3a). The pore size distribution was acquired by Barrett–Joyner–Halenda (BJH) method (Figure 3b). The results reveal a typical hysteresis loop of type IV, correlating with the characteristics of mesoporous materials.^20^ An SSA of 185 m^2^ g^–1^ and an average pore size of 9.5 nm are observed. A large surface area and mesoporous structure are important properties for a catalyst as they provide abundant catalytic active sites and diffusion channels for reactants, contributing to an enhanced ORR performance.^20,21,24^

**Figure 4:**
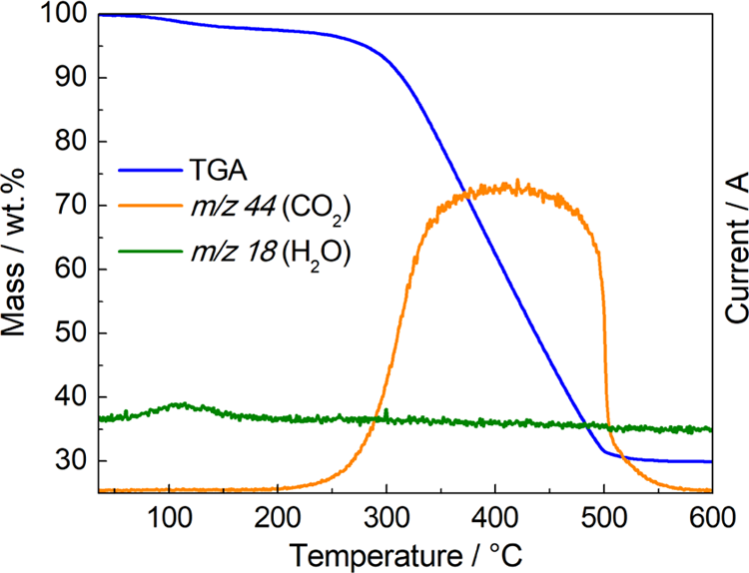
TGA-MS results of ZnCe-CMO/rGO-VC.

ICP-MS analysis was performed to determine the accurate metal concentration in the sample. The results show that 7.7, 5.1, 2.9, and 2.6 wt % of Mn, Ce, Co, and Zn, respectively, are present, resulting in a total metal content of 18.3 wt %, which also correlates well with the feed ratio. The remaining mass (approx. 80 wt %) is to a small content due to the oxygen content of the metal oxides present in the active material, and a large part is attributable to the support material, which is mainly composed of carbon and partly of the remaining oxygen-containing functionalities (e.g., hydroxyl, epoxy, and carboxyl groups) in the rGO material.^25^ The active material content (including oxygen) and the carbon support amount were determined by TGA-MS analysis. The sample was heated from ambient temperatures to 600 °C under an O_2_/Ar (20 vol %) atmosphere. The mass loss was recorded, and simultaneously the evolved gases were analyzed by MS. As can be seen in Figure 4, the thermal behavior of ZnCe-CMO/rGO-VC displays two characteristic mass changes between 35 and 600 °C.

After an initial almost constant region (35–100 °C), the water absorbed by the sample from the atmosphere evaporates at approx. 100 °C, as reflected by the *m*/*z* 18 signal (H_2_O^+^ evolution) obtained from the MS analysis. Thereafter, the mass remains barely constant (100–300 °C) again until the decomposition of carbon into CO_2_ (*m*/*z* 44 indicates CO_2_ evolution) takes place between 300 and 500 °C. Finally, the mass is stabilized at approx. 30 wt %, which can be directly related to the quantity of the active material, and in parallel, the quantity of the carbon support material is 70 wt %, correlating with the feed rates from the synthesis and the ICP-MS results.^18,26^

### Electrochemical Results of the ORR Catalysts

**Figure 5:**
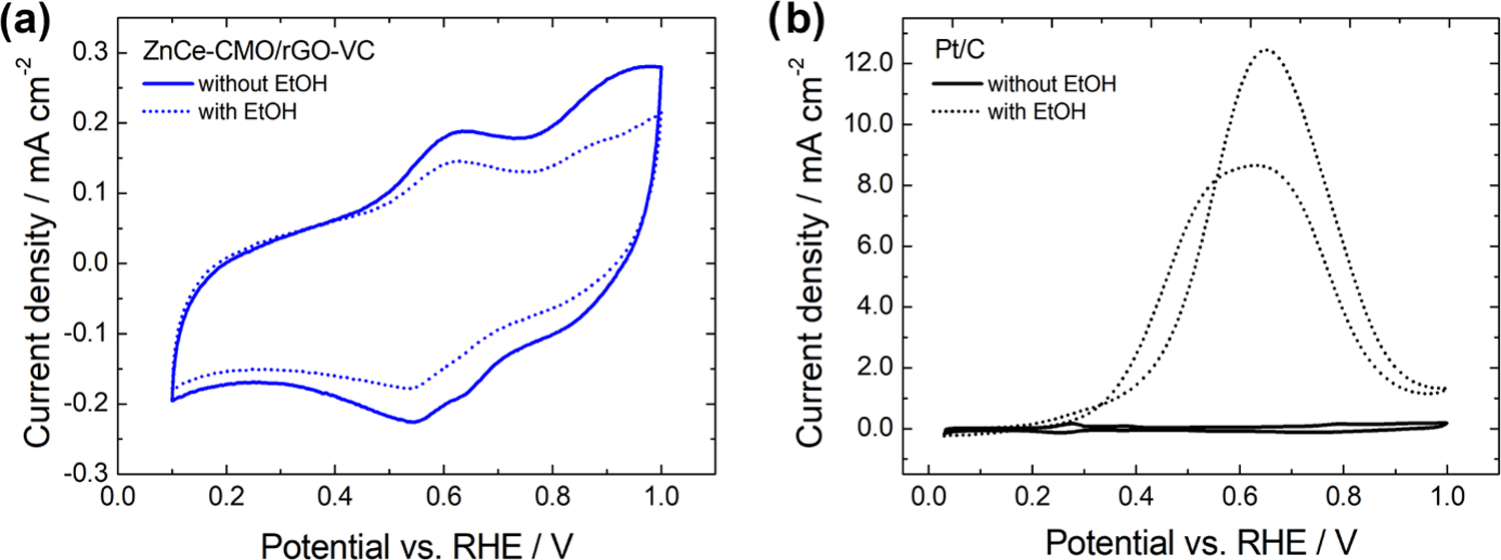
CV curves of (a) ZnCe-CMO/rGO-VC and (b) a commercial Pt/C in de-aerated 1 M KOH at 10 mV s^–1^ in the absence (compact line) and presence (dashed line) of 1 M EtOH.

The electrochemical properties of the ZnCe-CMO/rGO-VC catalyst were investigated by CV, LSV, and CA techniques and were compared with a commercial Pt/C catalyst. The CV results are shown in Figure 5.

The CV tests were performed to evaluate the oxidation and reduction processes in N_2_-purged 1 M KOH and were repeated in a mixture of 1 M KOH/1 M EtOH to examine the impact of ethanol. As can be seen in Figure 5a, similar redox profiles are observed for ZnCe-CMO/rGO-VC independent of the absence/presence of ethanol. A small drop in current density is noticed, however, which can be attributed to the minimal blocking of the active sites by ethanol.^7^ Nevertheless, it should be highlighted that the catalyst shows no activity toward the EOR compared to the commercial Pt/C (Figure 5b), which presents a large EOR peak. The redox peaks in the anodic and cathodic scan directions of the ZnCe-CMO/rGO-VC CVs can be related to Co and Mn reduction and oxidation processes as found for Co–Mn-containing spinels in the literature.^27−29^ Redox reactions of Zn and Ce do not proceed in this potential range.^19,21^ The oxidation peaks at approx. 0.62 and 0.92 V vs RHE can therefore be attributed to the Mn(II)/Mn(III) and Mn(III)/Mn(IV) redox couples that overlap with Co(II)/Co(III). The related reduction peaks are detected at approx. 0.65 and 0.54 V vs RHE.

**Figure 6:**
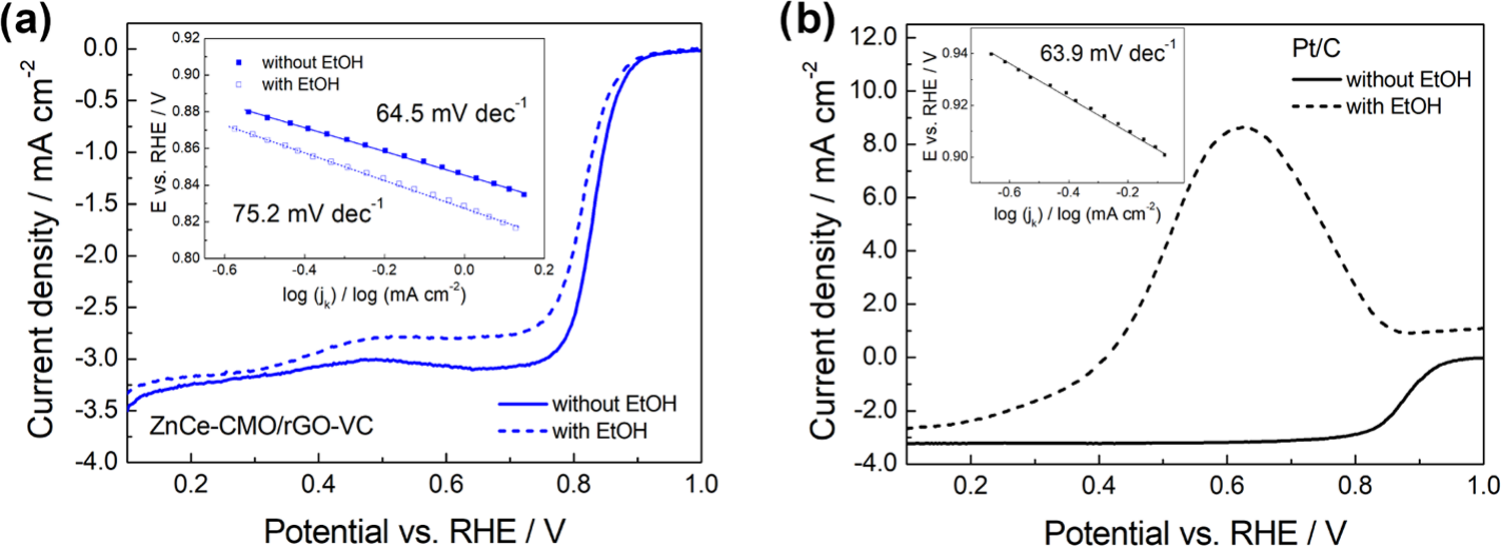
ORR polarization curves of (a) ZnCe-CMO/rGO-VC and (b) a commercial Pt/C at 1600 rpm in O_2_-saturated 1 M KOH at 10 mV s^–1^ in the absence (compact line) and presence (dashed line) of 1 M EtOH with Tafel plot insets.

The activity of ZnCe-CMO/rGO-VC toward ORR was evaluated by LSV experiments at 10 mV s^–1^ in O_2_-saturated 1 M KOH electrolyte solution, and the influence of EtOH on the ORR performance was determined in a mixture of 1 M KOH/1 M EtOH. The LSV curves of the composite at 1600 rpm and the corresponding Tafel plots (inset) are presented in Figure 6a, and the commercial Pt/C curves are shown in Figure 6b for comparison.

An excellent electrocatalytic ORR activity of ZnCe-CMO/rGO-VC in the absence of ethanol is highlighted by a diffusion-limited current density (*j*_D_) of −3.07 mA cm^–2^, an onset potential (*E*_onset_) at −0.1 mA cm^–2^ of 0.91 V vs RHE, a half-wave potential (*E*_1/2_) of 0.83 V vs RHE, and a Tafel slope of 64.5 mV dec^–1^ (Figure 6a). The values are close to that of the commercial Pt/C, which exhibits a *j*_D_ of −3.23 mA cm^–2^, an *E*_onset_ of 0.96 V vs RHE, an *E*_1/2_ of 0.87 V vs RHE, and a Tafel slope of 63.9 mV dec^–1^ (Figure 6b). The good catalytic performance of ZnCe-CMO/rGO-VC toward ORR can in general be attributed to the synergistic effects originated from the unique physicochemical properties of ZnCe-CMO nanoparticles well distributed and anchored on the carbon support material. The homogeneous distribution on the carbon material (as shown with SEM) ensures a large SSA (BET) and thus more active sites for the catalysis of the ORR. It was previously shown that the excellent conductivity of rGO additionally improves the interfacial charge transfer ability.^12,20^ In addition to the use of the carbon material, the good ORR performance is also provided by the outstanding properties of the different metal oxides in the active material and the synergistic effects between them. The ORR mechanism on the surface of TMOs is complex and still not fully understood. In general, it can occur via the direct 4e^–^ (eq 1) or more properly via the 2e^–^ (eqs 2 and 3) pathway on the surface cations of the TMOs. Both cases first involve the reduction of these cations, followed by adsorption of molecular oxygen on the active sites, and finally the formation of OH^–^ due to O–O bond cleavage (4e^–^ pathway) or the reduction of an OOH* intermediate to HO_2_^–^ (2e^–^ pathway).^4,30^ CMO has been employed as a promising ORR catalyst for many years due to its spinel structure and multiple valences.^8^ It was demonstrated in the literature that Mn cations rather than Co cations are considered as the catalytic active sites, as Mn^3+^ presents optimal ORR catalytic activity.^31^ Moreover, the properties of the CMO spinel can be tuned by modification with CeO_2_, which is due to the ability of CeO_2_ to act as an oxygen buffer, providing oxygen enrichment and activation.^19,32^ The additional use of ZnO is assumed to further optimize the catalytic behavior, as it has the ability to act as a forming agent, which leads to an increase in surface area and also tends to form oxygen vacancies.^21,33^ Thus, by adding ZnO, the *j*_D_ compared to a catalyst prepared in a previous study^19^ containing only Ce-modified CMO as an active material can be increased from −2.93 to −3.07 mA cm^–2^ and is comparable to the commercial Pt/C catalyst (−3.23 mA cm^–2^).

In the presence of ethanol, the polarization responses of ZnCe-CMO/rGO-VC toward the ORR are only slightly decreased, revealing that the catalyst is tolerant to ethanol. The value for *j*_D_ is reduced to −2.93 mA cm^–2^, the *E*_onset_ and *E*_1/2_ are shifted to 0.89 V vs RHE and 0.81 V vs RHE, respectively, and the Tafel slope is slightly higher at 75.2 mV dec^–1^ (Figure 6a). The ORR of the Pt/C, in contrast, is suppressed by a large EOR peak (Figure 6b). Therefore, the ZnCe-CMO/rGO-VC catalyst is selective and not susceptible to the formation of mixed potentials in the case of ethanol crossover during the cell operation, and a generally high performance can be ensured. This property can be a great advantage over state-of-the-art Pt/C catalysts, when used in, e.g., ADEFCs.

**Figure 7:**
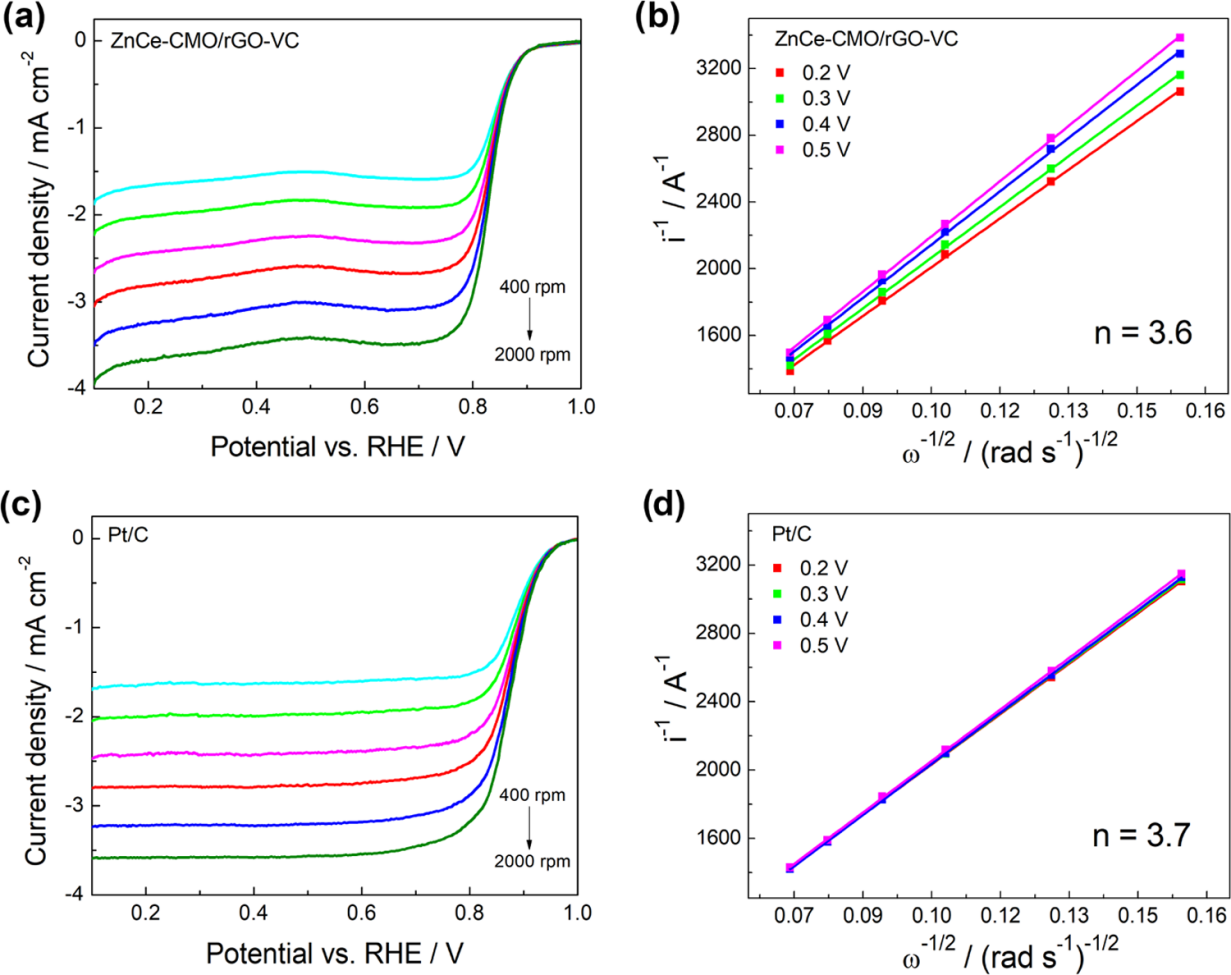
ORR polarization curves (left) at different rpm in O_2_-saturated 1 M KOH at 10 mV s^–1^ and corresponding Koutecky–Levich plots (right) of (a, b) ZnCe-CMO/rGO-VC and (c, d) Pt/C.

To further investigate the ORR mechanism, the LSV experiments in 1 M KOH electrolyte solution were carried out at different rotation rates (Figure 7a,c).

The RDE polarization curves were used to construct Koutecky–Levich plots (Figure 7b,d), and the electron transfer number (*n*) is calculated according to the Koutecky–Levich equation as follows,
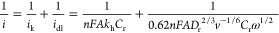
4where *i* is the measured current (A), *i*_k_ and *i*_D_ are the kinetic and diffusion-limited currents (A), respectively, *n* is the electron transfer number, *F* is the Faraday constant (96 485 C mol^–1^), *A* is the geometric electrode area (0.196 cm^2^), *k*_h_ is the electron transfer rate constant, *C*_r_ is the saturated O_2_ concentration in 1 M KOH (7.8 × 10^–7^ mol cm^–3^), *D*_r_ is the diffusion coefficient of O_2_ in 1 M KOH (1.8 × 10^–5^ cm^2^ s^–1^), *v* is the kinematic viscosity (0.01 cm^2^ s^–1^), and ω is the rotation speed of the electrode (rad s^–1^).^34,35^ The ORR can proceed via either the favored 4e^–^ process (eq 1), where O_2_ is directly reduced to OH^–^ or the inhibited 2e^–^ process (eqs 2 and 3) including HO_2_^–^ intermediate formation.^21^ The ZnCe-CMO/rGO-VC exhibits a high electron transfer number (*n* = 3.6) comparable to that of the commercial Pt/C (*n* = 3.7), indicating that four electrons are transferred in the oxygen reduction reaction pathway.

**Figure 8:**
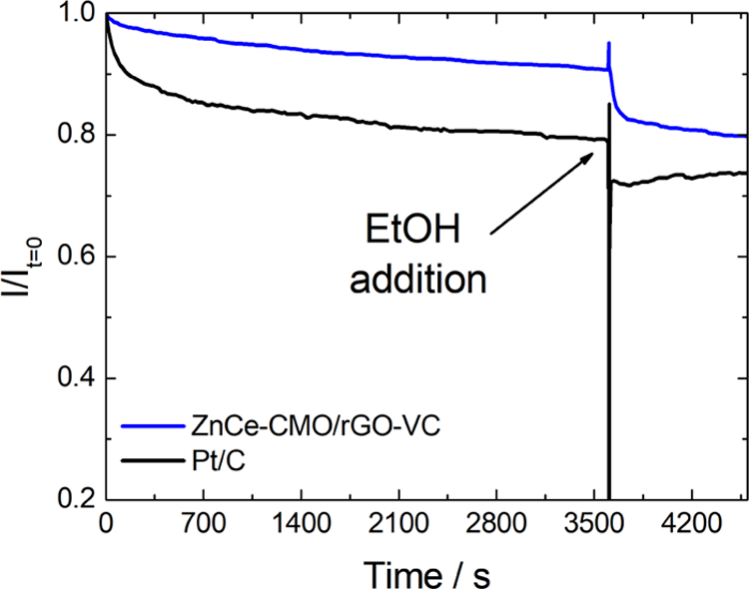
CA of ZnCe-CMO/rGO-VC at 0.4 V vs RHE in O_2_-saturated 1 M KOH at 1000 rpm with EtOH addition after 3600 s.

CA tests were conducted in O_2_-saturated 1 M KOH electrolyte solution to examine the stability of ZnCe-CMO/rGO-VC for 3600 s at 0.4 V vs RHE at 1000 rpm, and the impact of EtOH was examined after rapid EtOH addition at 3600 s. As can be seen in Figure 8, the catalyst presents excellent stability with a higher remaining current density of 91% compared to the value for the commercial Pt/C (79%), which is also found in the literature to be between 70 and 80%.^12,19,33,35^

The improved stability is mainly attributed to the synergistic effects between ZnO, CeO_2_, CMO, and carbon support, and the expected strong C–O–metal interactions between rGO and the metal oxides as shown previously in the literature.^16,21^ The rGO material still retains important oxygen-containing functional groups on its surface, such as epoxy or hydroxyl, which promote the interfacial interaction between the TMOs and the carbon support. As shown by Zhou et al.,^36^ the strong coupling is induced by the oxygen bridges, which mainly originate from the pinning of the epoxy/hydroxyl groups on the metal atom. This phenomenon prevents the particles from agglomerating and detaching, thus ensuring high stability.

In addition, it is observed that the current density of the ZnCe-CMO/rGO-VC is slightly decreased due to poisoning of the active sites after the injection of EtOH and is still retained at 80% after 4600 s. In comparison, the Pt/C shows a large fluctuation during the EtOH addition, which is attributed to the EOR response of Pt. Though long-term stability testing would offer further insight, the results of this study demonstrate that the prepared ZnCe-CMO/rGO-VC catalyst is not only very active for ORR but also displays high short-term stability and selectivity and can therefore be a promising ORR catalyst.

## Conclusions

A novel nanocomposite of Zn- and Ce-modified cobalt-manganese oxide (CMO) anchored on reduced graphene oxide (rGO) and Vulcan carbon (VC) was synthesized for use as a highly active, stable, and selective ORR catalyst in alkaline media. The results show that ZnCe-CMO/rGO-VC exhibits unique physicochemical properties with evenly distributed metal oxide nanoparticles on the carbon support material with a mesoporous morphology and a high SSA (185 m^2^ g^–1^). Electrochemical tests resulted in an excellent activity toward ORR with a *j*_D_ of −3.07 mA cm^–2^, an *E*_onset_ of 0.91 V vs RHE, an *E*_1/2_ of 0.83 V vs RHE, and an *n* value of 3.6. The tests in the presence of ethanol revealed that the catalytic activity is scarcely affected by the presence of ethanol when compared with a commercial Pt/C catalyst, as the catalyst shows no EOR activity, highlighting ZnCe-CMO/rGO-VC as a selective ORR catalyst material. Besides the good ORR activity and ethanol tolerance, superior stability is observed in the CA test, resulting in a remaining current density of 91% after 3600 s and of 80% after EtOH addition. The enhanced ORR performance could be attributed to abundant active and stable catalytic sites and fast charge transfer induced by synergistic effects between metal oxides and rGO support materials. The obtained results suggest that the ZnCe-CMO/rGO-VC nanocomposite is a promising non-precious-metal alternative for boosting ORR in alkaline fuel cells with high activity, stability, and selectivity in alkaline electrolytes.
